# Robotic segmentectomy for early-stage lung cancer

**DOI:** 10.3389/fsurg.2023.1090080

**Published:** 2023-03-23

**Authors:** Elisabeth Savonitto, Kazuhiro Yasufuku, Alison M. Wallace

**Affiliations:** ^1^ Division of Thoracic Surgery, Queen Elizabeth II Health Sciences Centre, Dalhousie University, Halifax, NS, Canada; ^2^ Division of Thoracic Surgery, Toronto General Hospital, University of Toronto, Toronto, ON, Canada

**Keywords:** lung cancer, robotic-assisted surgery, non-small cell lung cancer, lobectomy, segmentectomy

## Abstract

Lobectomies have long been the gold standard for surgical treatment of early-stage non-small cell lung cancer (NSCLC), with segmentectomies limited to instances of benign disease or as an alternative in patients where lung preservation is indicated. However, a recently published randomized control trial has demonstrated the superiority of segmentectomy over lobectomy in terms of overall survival for early-stage lung cancer. Segmentectomy could thus be considered a standard procedure for small-sized peripheral NSCLC. While segmentectomy *via* video-assisted thoracic surgery (VATS) is the most widespread approach, development in video instrumentation and thoracic robotic surgery is rapidly gaining interest. Indeed, robotic surgery pioneers boast the advantages in three-dimensional view, improved magnification, ergonomics, dexterity, safety, and ease of surgery with this technology. This review aims to outline robotic-assisted segmentectomy indications, preoperative evaluation, and the operative conduct for the different lung segments from a single surgeon console. There are many ways to perform segmentectomies and therefore this review describes generalized approaches that can be tailored based on experience.

## Introduction

Since the Lung Cancer Study Group published the prospective randomized controlled trial in 1995 demonstrating a three-fold increase in locoregional recurrence and an increase in mortality after sublobar resection for T1N0 non-small cell lung cancer (NSCLC), lobectomies have been the gold standard for surgical treatment of early-stage NSCLC. Segmentectomy, defined as the anatomical segmental resection with sufficient margins (≥2 cm or ≥ the size of the nodule) and lymph node assessment (N1 and N2 lymph node stations) (1), have since then been limited to benign disease or as an alternative to lobectomy in patients with limited cardiopulmonary reserve or other major comorbidities (2, 3). The goal of these sublobar resections is to preserve lung parenchyma and therefore pulmonary function, with the thought that this would reduce postoperative morbidity while achieving the primary therapeutic goal of the surgery (4). More recently, with the increase in computed tomography (CT) screening in patients at high risk for lung cancer, in addition to advances in imaging technology, early detection of smaller but none-the-less concerning peripheral lung nodules has increased in frequency. All of which raises the question of whether or not sublobar resection, or more specifically segmentectomy, could be the standard of care for peripheral early-stage NSCLC (2, 3). Up until recently, only retrospective studies have shown that in carefully selected populations, segmentectomy demonstrated oncological results comparable to lobectomy (5, 6).

Consequently, the West Japan Oncology Group and Japan Clinical Oncology Group recently published a multicentered randomized controlled trial (JCOG0802/WJOG4607L) and demonstrated the superiority of segmentectomy over lobectomy in terms of overall survival for early-stage lung cancer. This study included patients with clinical stage IA NSCLC (peripheral tumours; tumour diameter ≤2 cm; consolidation-to-tumour ratio >0.5). Reportedly, the 1,106 patients studied in Japan showed an improved 5-year overall survival in the segmentectomy group (94.3% in the segmentectomy group compared to 91.1% in the lobectomy group, *p* < 0.0001 for non-inferiority and *p* = 0.0082 for superiority), with an overall improved survival across all subgroups, especially in men, patients older than 70 years old, solid tumours as well as non-adenocarcinomas in the segmentectomy group. Additionally, the 5-year relapse-free survival was 88.0% in the segmentectomy group and 87.9% in the lobectomy group (HR: 0.998, 95% CI: 0.753–1.323). However, they noted a statistically significant increase in locoregional relapses in the segmentectomy group (11% vs. 5%, *p* = 0.0018), explained by the lesser resection in segmentectomy, and we propose limited knowledge of lymphatic drainage of the lung, but somehow a nonsignificant difference on the total relapse pattern. Of the patients with relapses, the 5-year survival was higher in the segmentectomy group (68% vs. 49%) and a higher percentage of the relapses in the segmentectomy group received treatment compared to the lobectomy group (93% vs. 80%). As expected, there was significant difference in the reduction in forced expiratory volume in 1 s (FEV1) between segmentectomies and lobectomies, which was higher in the lobectomy group; however, the proportions of median FEV1 reduction between segmentectomy and lobectomy groups at the 6 and 12 months follow-up were not clinically significant (4, 5).

The JCOG0802/WJOG4607L study is the first randomized-controlled trial to demonstrate the non-inferiority and superiority of segmentectomy over lobectomy for clinical stage IA NSCLC overall-survival and could thus be considered a standard procedure for small-sized peripheral NSCLC. While segmentectomy *via* video-assisted thoracic surgery (VATS) is the most widespread approach, development in video instrumentation and thoracic robotic surgery has gained more and more interest. Indeed, robotic surgery pioneers boast the advantages in three-dimensional view, improved magnification, ergonomics, dexterity, safety, and ease of surgery, especially with regards to the mediastinal lymph node dissection, with such technology (7–9). Furthermore, adjuncts such as the systemic injection of indocyanine green have been shown to enhance the demarcation of intersegmental plane facilitating segmentectomies (10).

**Figure 1:**
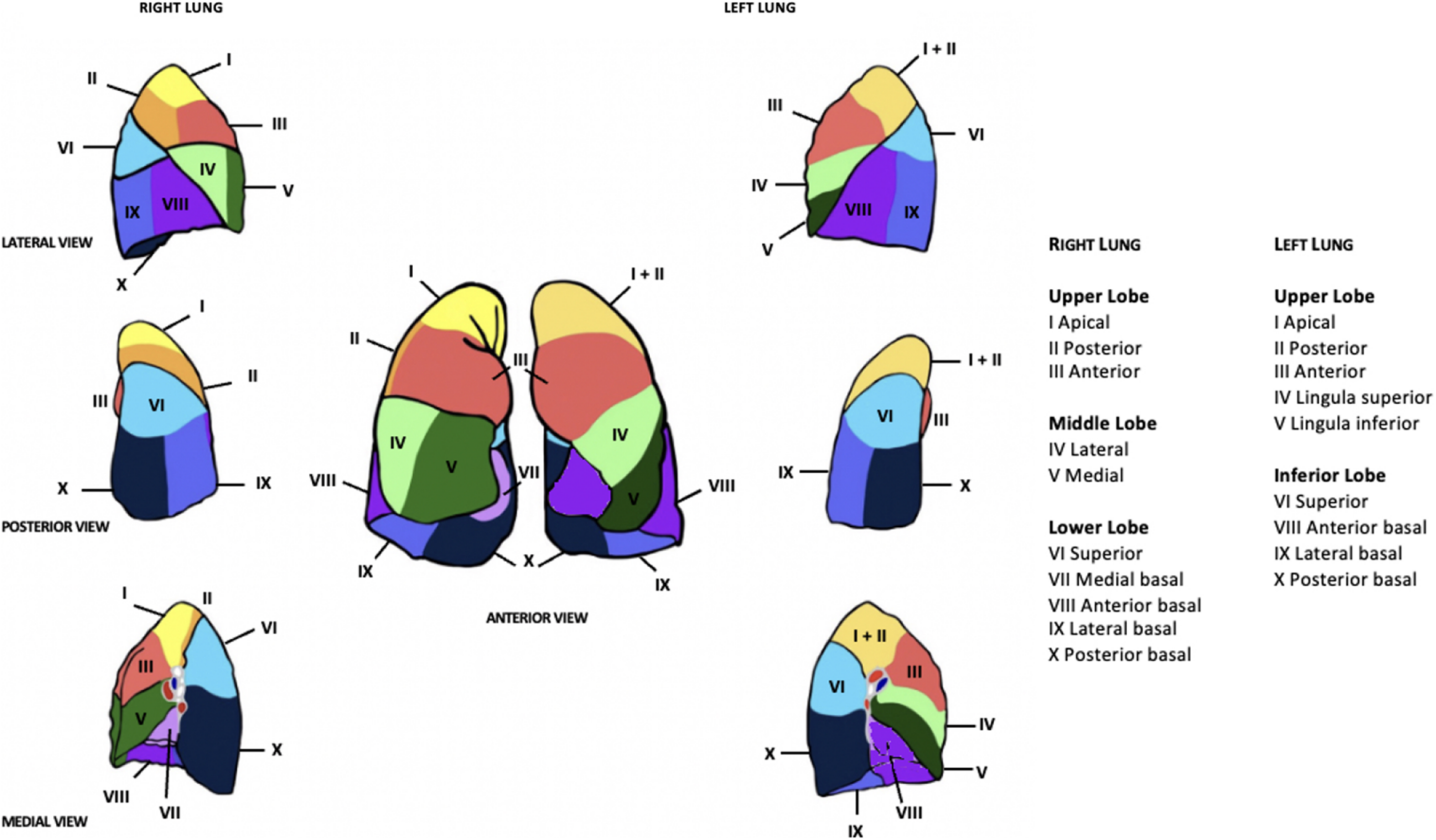
Lung segments by Elisabeth Savonitto.

**Figure 2:**
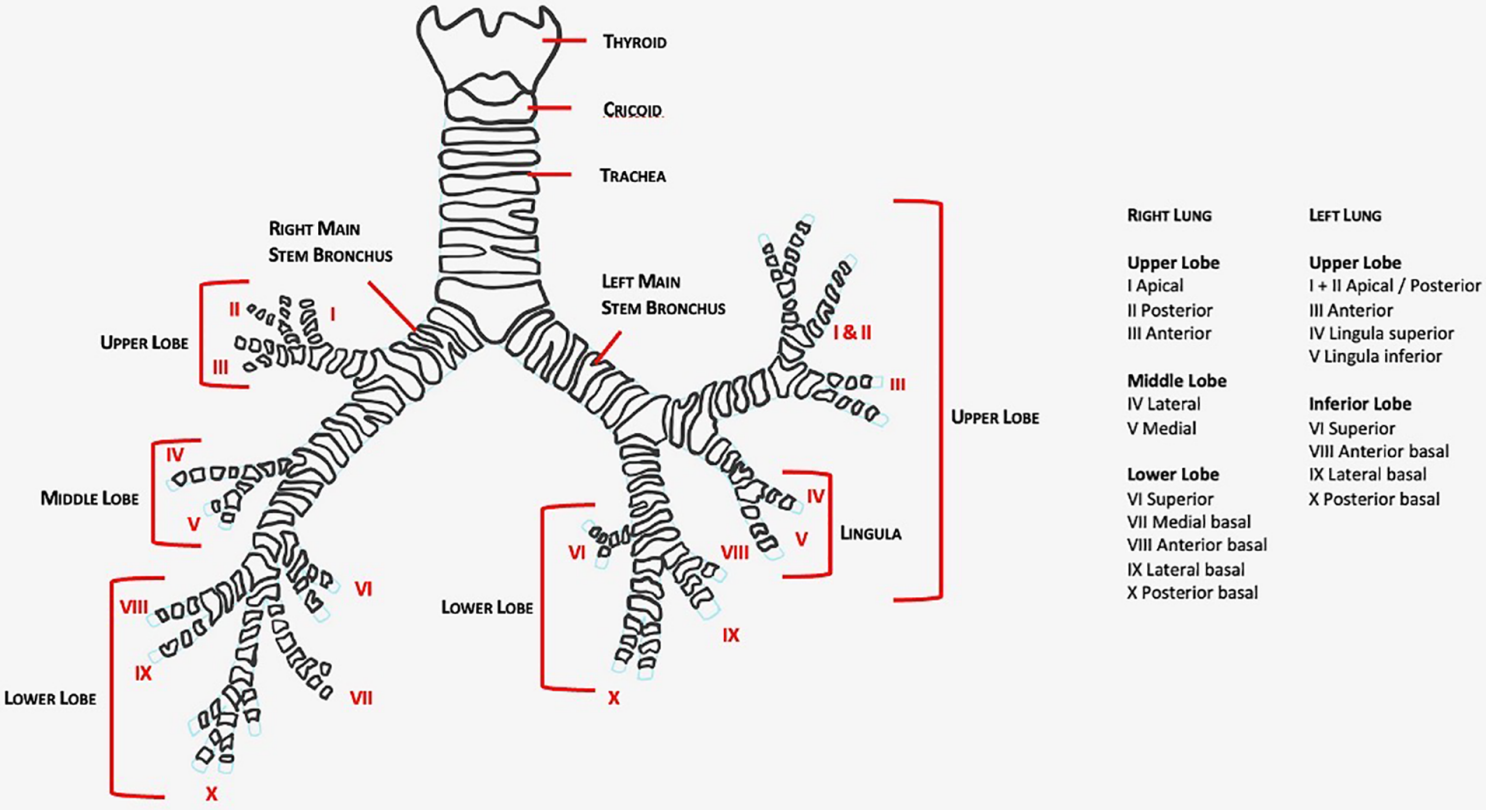
Bronchial tree by Elisabeth Savonitto.

This article aims to review robotic-assisted segmentectomy indications, preoperative evaluation, and the operative conduct for the different segments from a single surgeon console. There are many ways to perform segmentectomies and therefore this article outlines a generalized approach that can be tailored based on experience. The anatomy of the lung segments is outlined in Figures 1, 2.

## Indications and preoperative considerations

The standard preoperative evaluation of early-stage lung cancer including a clinical assessment, PET-CT and pulmonary function testing apply. In order to accurately select patients who will benefit from segmentectomy, it is important to stage the mediastinum *via* endobronchial ultrasound transbronchial needle aspiration (EBUS-TBNA) or video mediastinoscopy as segmentectomy is not indicated if there is lymph node involvement according to the JCOG0802/WJOG4607L study (3). Additional imaging and testing should be organized according to patient past medical history and suspicion for metastasis (11, 12).

Robotic surgery of any kind requires a dedicated team of surgeons, nurses and support staff who are trained in robotic-assisted surgery. In thoracic surgery, plans need to be in place and simulations run in preparation for the event to convert to thoracotomy.

## Robotic console adjustments

The Food and Drug Administration (FDA) has approved the Da Vinci Surgical Systems (Intuitive Surgical Inc., Sunnyvale, CA, USA) for robotic anatomic lung resections with several new platform developments on the horizon. With the Si system, the robot is driven from the head of the patient, over the shoulder, forming a 15-degree angle with the patient length. The anesthesia team provides care on the backside of the patient, 90-degrees from the console, and often require the use of longer ventilation tubing as they are a further distance away than their typical location. For the Xi system, the robot can approach the patient at a 90-degree angle from the side of the bed, allowing anesthesia to be in their standard position at the head of the patient. Special attention confirming the tolerance of one-lung ventilation prior to sterile drape placement is imperative as it is difficult to reposition the endotracheal tube once the robot is docked.

## Patient positioning and port placement

**Figure 3:**
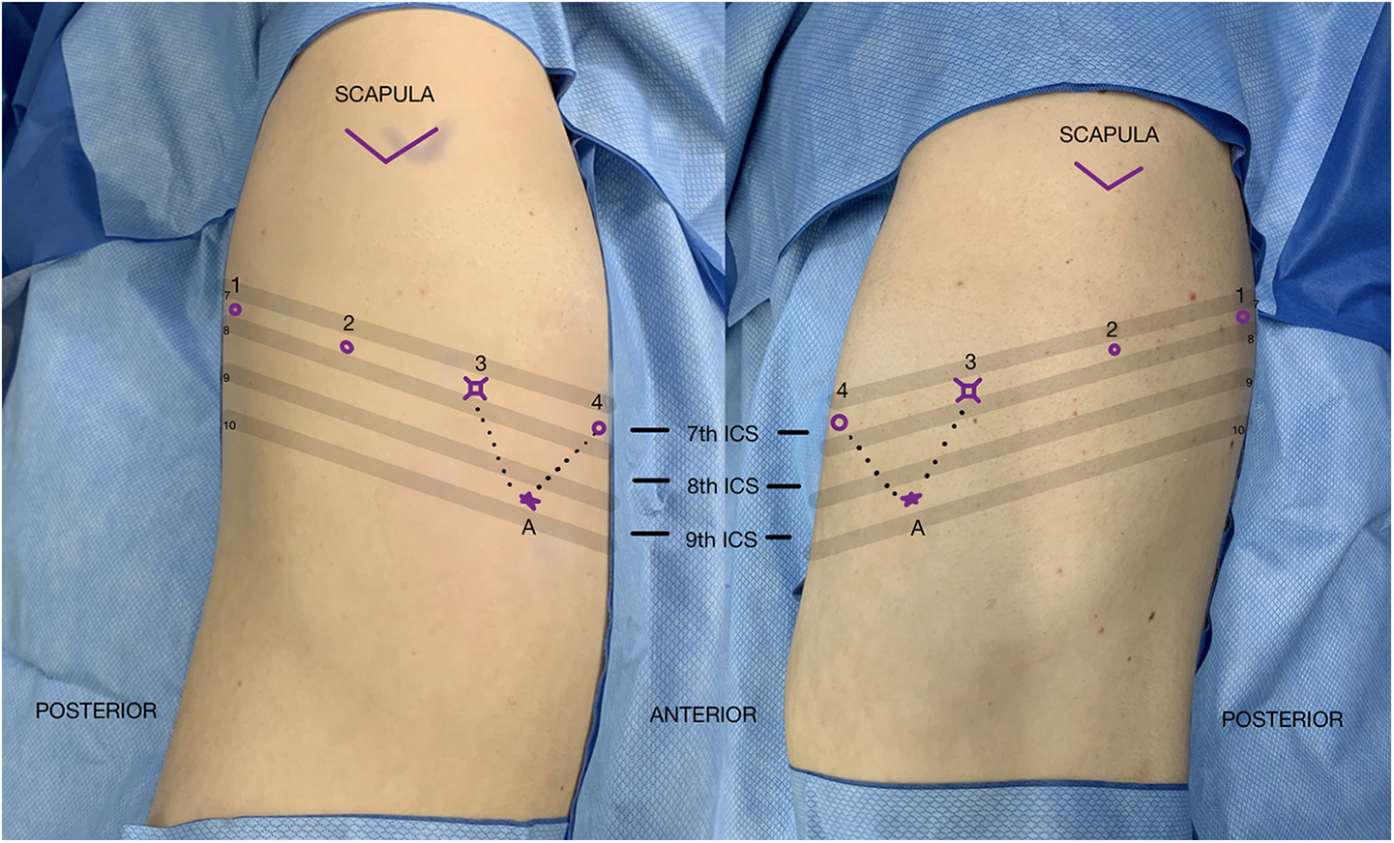
Port placement for robotic segmentectomy by elisabeth savonitto.

Patient positioning is similar to a VATS segmentectomy: the patient is placed in lateral decubitus position, with the surgical side up. The table is flexed 5–10 degrees at the level of xiphoid process to open the intercostal space maximally. Following flexion, the bed is adjusted to level the chest and the bed height is set to as low as possibleThe patient is then secured in position with the pressure points checked and padded. The patient is then prepped and draped in the usual sterile fashion, allowing sufficient exposure if there was a need for conversion to thoracotomy. Port placement is surgeon dependent based on previous experience and personal preference. Figure 3 depicts port placement for a robotic segmentectomy using four robotic arms in the same intercostal space with an assistant port triangulated between ports 3 and 4. The initial camera port is placed in the 7th intercostal overtop the 8th rib between the mid axillary line and anterior axillary line. The hemithorax is inspected in order to optimize further port placement. Ports 1 and 2 are placed posterior to port 3 and port 4 is placed anterior to port 3 all in the same intercostal space maintaining 6 cm–10 cm between ports. The most posterior port, port 1, is placed at minimum of 4 cm anterior to the spine. One variation is to place the most anterior port, port 4, in the 6th intercostal space, The assistant port is triangulated between ports 3 and 4 at the junction of the diaphragm and chest wall.

Similar to VATS, carbon dioxide insufflation (5–8 mmHg pressure at 4L/min flow rate) can be used to depress the diaphragm and compress the lung, but is not mandatory. A zero-degree camera is most commonly used for these operations but based on surgeon preference a 30-degree camera can be used as well.

## Mediastinal lymph node dissection

As with any cancer operation, robotic segmentectomy begins with inspection of the pleura to rule out any sign of metastatic disease. According to the JCOG0802/WJOG4607L study systematic or selective lymph node dissection was mandatory, and nodal sampling was not allowed (3).

Starting with the mediastinal lymph node dissection has several advantages. First, removal of the lymph nodes helps expose the hilar structures. Second, sending off the lymph nodes for pathologic analysis at the start of the case allows the pathologist time to analyze the tissue to rule out metastatic disease. As the recent findings for the superiority of segmentectomies compared to lobectomies apply to T1N0 disease, conversion to lobectomy would be indicated if the lymph nodes were positive on frozen section analysis.

Division of the inferior pulmonary ligament allows exposure of lymph node stations 8 and 9, which are then carefully dissected and removed. With the accessory arm, the lower lobe is retracted medially and anteriorly to expose station 7, which is then dissected and removed.

Regarding lymph node stations 2R and 4R, the right upper lobe is retracted inferiorly with the accessory arm. Regarding the left-sided resections, the accessory arm is used to press caudally the left upper pole to expose lymph node stations 5 and 6. Attention is paid to avoid injury to the recurrent laryngeal nerve.

## Right upper lobe apical segmentectomy

One approach is from the anterior hilum, with posterior retraction to the lung with the accessory arm. The tissue between the apical artery and vein is dissected. The upper lobe vein is then anatomized until its first branch, the apical vein, is apparent. Dissecting the vein first exposes the upper lobe arteries. It is key to expose the bifurcation between the apical and anterior lobe arteries to then dissect the apical artery. The artery is then stapled, followed by the vein. This will expose the B1 bronchus branch which can be isolated and stapled. The use of a vessel-loop can aid to pass staplers around the vessels and bronchi. The parenchymal segmental delimitation can be facilitated with either re-insufflation of the lung or with injection of indocyanine green and use of the “firefly view” to assess areas free of blood flow. The parenchyma can then be safely divided with a surgical stapler (13).

## Right upper lobe posterior segmentectomy

The posterior approached dissection of the lymph nodes will help differentiate the posterior ascending artery from the superior lobar arteries. Note that this artery is absent in 10%–15% of the population. Once dissected and defined, it can be stapled. Then the fissure between the superior and inferior lobe is dissected, which will further expose the right main bronchus, which can be followed until exposure of the posterior segmental bronchus of the right upper lobe. The posterior branch of the right upper lobe can then be dissected. The posterior segment parenchyma can then be separated from the remnant upper lobe with a stapler.

## Right upper lobe anterior segmentectomy

An anterior vein-first approach will facilitate the anatomy exposure. The anterior hilum is incised at the level of the right superior pulmonary vein, which is then followed until its trifurcation. The anterior segmental vein typically has a more oblique and anterior orientation. There can be multiple branches. This will then expose the superior lobar artery and its division. The anterior segmental artery has a more oblique and downward orientation towards the anterior segment parenchyma; it can then safely be stapled with a vascular load once dissected out. As the posterior vein travels between the anterior and posterior bronchus, following it will help landmark the anterior bronchus, which can then be stapled. Finally, the parenchyma can be transected in the usual fashion (14).

## Superior segmentectomy of the left lower lobe

By retracting the lung anteriorly, the fissure can be dissected freely posteriorly, which will expose the bifurcation of the left pulmonary artery. Following the inferior lobar artery will expose the lingular artery and the basal trunk. The superior segmental artery can then be seen more medially and divided. The superior segmental vein will then appear to the surface and can also be divided. Finally, with either an anterior or posterior approach, the superior segmental bronchus will be seen just distal to the left main bronchus bifurcation; it can then be divided. Reinflating the lung or using the Firefly view will help demarcate the superior segment from the rest of the lower lobe and can then be transected, which is usually done through the assistant port.

## Basilar segmentectomy

An anterior vein-first technique, while pulling the lung posteriorly, is the preferred approach for the basilar segmentectomy. The basilar segmental veins are isolated and stapled, by following the inferior pulmonary vein and preserving the superior segmental vein, which are usually more proximal. This will then expose the bronchus to the basilar segments, which can then be divided, with caution to preserve the superior segmental bronchus that usually has a more medial direction. The fissure can then be dissected completely, which will expose the arteries. The basilar segmental arteries can then be anatomized and divided accordingly. The parenchyma is then stapled as previously described, while preserving the superior segment of the lower lobe (11). Each of the basilar segments can be transected individually. In order to ensure division of the appropriate structures, the safest approach is to start from the peripheral parenchyma and progress medially.

## Left upper apical trisegmentectomy

Anteriorly, the mediastinal pleura overlying the superior pulmonary vein is sharply incised and is bluntly dissected until exposing the inferior pulmonary vein. Posteriorly, the area between the upper and lower lobes is dissected to expose the bifurcation between the superior and inferior left pulmonary arteries. The first branches consist of the apical and posterior superior arteries, which can then be divided. The left main pulmonary artery is anatomized in a way to expose the anterior branches that pass under the aortic arch. This will expose the left superior pulmonary vein which can be dissected and followed. Its bifurcation will expose the trisegment pulmonary vein. The arteries and veins can then be stapled. Finally, their dissection will expose the bronchus. the left main bronchus is followed until its bifurcation, which will again separate to expose the superior upper bronchus and the lingular branches. The apicoposterior division can then be stapled. The segmentectomy is completed by stapling the parenchyma between the lingula and the apical trisegment, which can be demarcated as previously described (15).

## Lingulectomy

**Figure 4:**
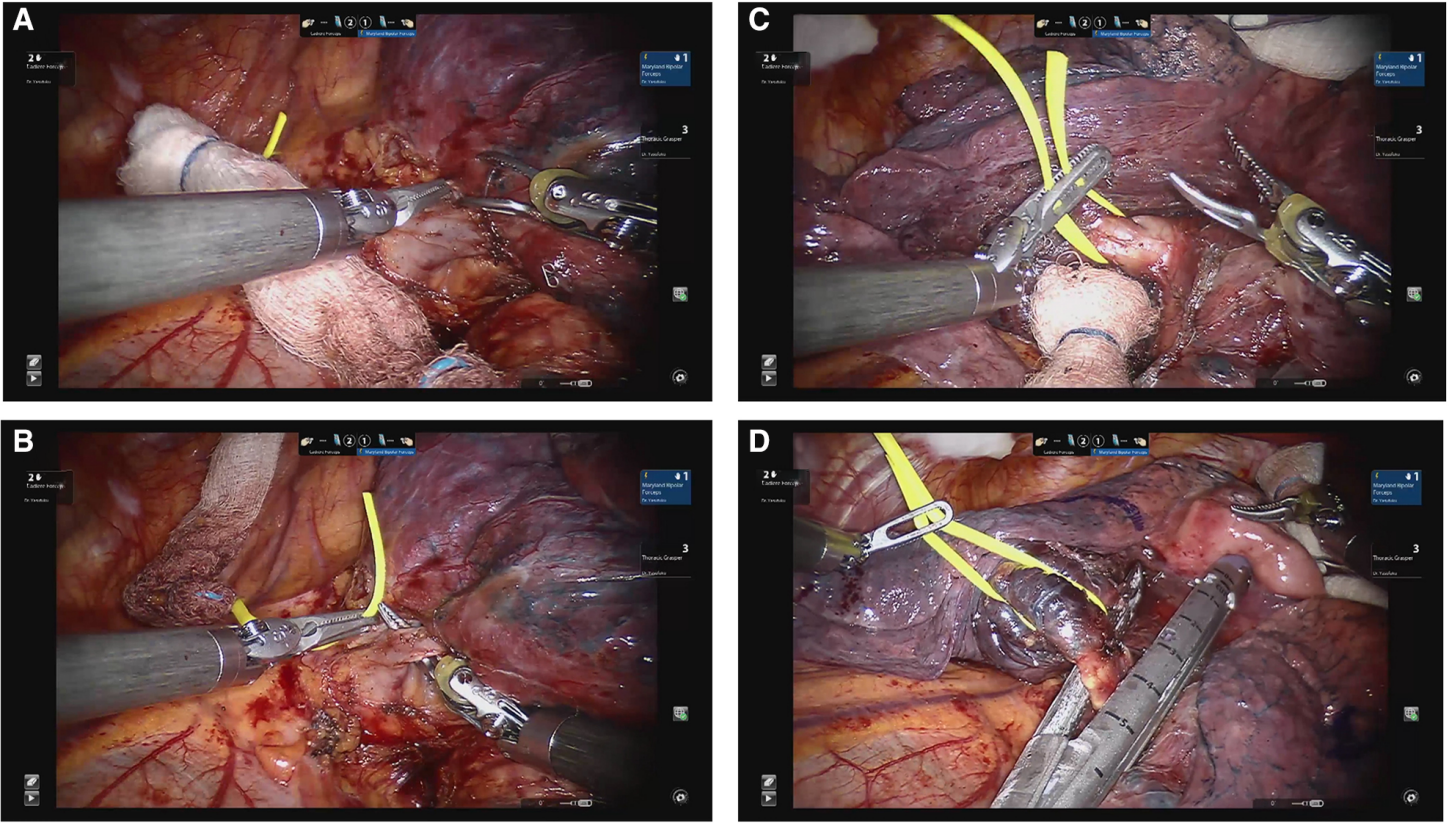
Lingulectomy. (**A**) Dissection of lingular vein. (**B**) Encircling lingular vein. (**C**) Dissection of lingular PA branches. (**D**) Division of lingular bronchus.

Intra-operative figures of a robotic-assisted segmentectomy are shown in Figure 4. Using the same technique as the left upper apical trisegmenctecomy, the fissure between the upper and lower lobes is followed and divided while approaching through the posterior aspect of the lung, which then exposes the inferior lobar artery, which is followed until the first branches, the lingular artery or arteries, are exposed. They can then be divided with the assistant port or the left arm. The superior pulmonary vein will then come to the surface and will be followed until the bifurcation between the apical and lingular branches are exposed. The lingular branches are then divided through the assistant port. Finally, the left main bronchus will be followed until the lingular bronchus are anatomized, to then be divided. Finally, the parenchyma is stapled.

## Postoperative management

Following segmentectomy, the average length of stay is 3–5 days (16). Pain management protocols recommended for VATS lobectomies can be employed for segmetectomies including, regional analgesic techniques such as a single shot paravertebral block or intercostal nerve blocks along with systemic analgesia including Tylenol and non-steroidal anti-inflammatory drugs or cyclo-oxygenase-2-specific inhibitors administered pre-operatively and continued postoperatively (17). Opioids are used as rescue analgesics postoperatively (17). Furthermore, adopted from VATS lobectomies, one 20-F or 24-F chest tube directed apically with minimal chest tube suction is recommended (18). The chest tube is removed when there is no air leak and the daily output is less than 450 ml (non-chylous and non-sanguinous) (18). I COUGH, a standardized perioperative pulmonary care program, can be employed to improve patient performance and reduced pulmonary complications (19).

## Concluding remarks

In conclusion, the recently published randomized controlled trial JCOG0802 confirmed the superiority and non-inferiority of segmentectomies in context of peripheral T1N0 NSCLC. Many techniques have been described, and robotic-assisted thoracic surgery is on the rise. This article describes robotic-assisted segmental resections. One of the incredible things about lung cancer surgery is that although cases may start with a general approach they often unravel as unique and intricate puzzles and no matter what approach is used initially it is important to be adaptable and familiar with the tools available.

## Author contributions

ES and AW conceived the concept and wrote the manuscript, and KY made substantial, direct intellectual contributions to the work. All authors contributed to the article and approved the submitted version.

## Conflict of interest

The authors declare that the research was conducted in the absence of any commercial or financial relationships that could be construed as a potential conflict of interest.
